# 84. Eosinophilic fasciitis

**DOI:** 10.1093/rap/rky034.047

**Published:** 2018-09-20

**Authors:** Janardhana Golla, Emma J Robinson

**Affiliations:** 1 Rheumatology, NHS Tayside, Dundee, UNITED KINGDOM; 2 Gastroenterology, NHS Tayside, Dundee, UNITED KINGDOM

**Introduction:** Eosinophilic fasciitis is a rare disorder of unknown etiology and pathogenesis characterised by symmetrical erythema and oedema of extremities in early phase, followed by collagenous thickening of the subcutaneous fascia in the later stage. It was first described in 1974 by Shulman (known as Shulman syndrome) in two men with a scleroderma-like disease of the extremities associated with eosinophilia and hypergammaglobulinaemia. It was later named as eosinophilic fasciitis by Rodnan et.al based on striking histological features showing inflammation of fascia between the subcutis and the muscle. Eosinophilic fasciitis predominantly affects forearms and spare fingers. Absence of Raynaud’s and visceral manifestations are in contrast with scleroderma.

**Case description:** A 57-year-old bus driver was admitted to acute medical admissions for the third time over a three-month period with gradually progressive painful, swollen extremities. His legs were painful and heavy when he was on holiday in France and had done a lot of cycling. He initially noticed pain and swelling of his legs (calves) and ankles. The pain and tightness progressed to the extent that he had difficulty in climbing stairs. He also had intermittent wrist and bilateral hip pain with sparing of other joints. He developed painful, tight forearms two months after his leg symptoms. He had an itchy, nodular rash with plaques on his back for two years. His other symptoms included night sweats, weight loss, fatigue and dry cough. His past medical history included endoscopic nasal polypectomy four months prior to his symptoms and recent onset testicular pain with ultrasound showing bilateral varicoceles. He stopped smoking 25 years prior to his presentation and drank 24 units of alcohol a week. There was family history of colonic cancer (father), throat cancer (mother), lung cancer and Charcot Marie tooth disease (sister). His bowel cancer screening colonoscopy has shown haemorrhoids and diverticular disease. On examination he had swollen, erythematous forearms and legs with indurated skin. His proximal leg muscle power was slightly reduced secondary to pain. Systemic examination was normal. His blood counts showed eosinophilia (1.9 x 10^^9^ /L) with an elevated CRP (42) and a low albumin of 26. His eosinophils and CRP were mildly elevated in the nine months prior to his reported symptom onset. His liver function tests, urinalysis and echocardiogram were normal. CT chest, abdomen and pelvis was unremarkable. His ANCA, ANA, ENA and rheumatoid factor were negative with normal complements and negative Borrelia antibody screen. A total IgE was within normal range with negative Aspergillus antibody. Viral hepatitis and HIV tests were negative. Immunoglobulins showed normal IgG and IgA with a low IgM with a normal electrophoresis. MRI forearms showed extensive subcutaneous oedema with contrast enhancement of intermuscular and compartmental fascia consistent with a florid fasciitis. A full thickness forearm biopsy showed thickening of subcutaneous fascia with intense perivascular infiltration of lymphocytes with a very occasional eosinophil. He was initially treated with NSAIDs and noticed very limited improvement in his symptoms. He was later commenced on prednisolone 40mg daily after the forearm biopsy was taken. There was significant improvement in his symptoms with steroids. He was commenced on methotrexate and prednisolone dose was tapered. He had recurrence of pain and swelling when the prednisolone dose was less than 20mg. We increased the methotrexate dose to 25mg weekly and the prednisolone was tapered more slowly. We managed to wean his prednisolone gradually to 10mg daily over nine months without any recurrence of symptoms. His last eosinophil count and CRP were within normal limits.

**Discussion:** This previously fit and healthy gentleman had presented on three occasions with worsening painful swelling of his limbs. His initial investigations including urinalysis, liver function tests and echocardiogram were aimed at looking for a cause of peripheral oedema and low albumin. Due to uncertainty of diagnosis a number of immunology and other blood tests were performed. He was referred after his third admission when the above investigations failed to yield a diagnosis. Even after a careful history and clinical examination in the rheumatology outpatient clinic Eosinophilic fasciitis was not on our initial list of differentials. We initially also pursued a possible paraneoplastic myositis. It was only after his forearm MRI was reported as showing fasciitis that we began to consider Eosinophilic fasciitis. Unlike other reported cases in the literature he did not have a hypergammaglobulinaemia. His initial marked improvement with high dose steroids was encouraging, but unfortunately this was not sustained once the steroid dose was tapered. It has been challenging to manage his disease given the relapsing symptoms with the initial steroid wean. Subsequent changes to his steroid sparing DMARDs have been successful and we are now nearly at a point that we can stop steroids altogether. His improved disease control had beneficial effects on his physical activity including cycling. Interestingly whilst in the majority of cases no cause is found, intensive exercise has been linked to onset of this disease. Other possible triggers include haemodialysis, autoimmune and haematological conditions. This case is particularly interesting due to the rarity of the diagnosis and the classical examination finding of the groove sign on his forearms. His MRI images are very interesting and along with the biopsy results clinched the diagnosis. His clinical course on treatment has been somewhat stormy, but we are satisfied that he is now making good progress.

**Key Learning Points:** Eosinophilic fasciitis should be suspected in patients who present with erythema, swelling and induration of the extremities along with peripheral eosinophilia without systemic involvement. Presentation can be similar to systemic sclerosis but differs with sparing of the hands. Diagnosis is made with MRI showing fasciitis and full thickness biopsy showing thickening of subcutaneous fascia with intense perivascular infiltration of lymphocytes. Treatment is with immunosuppression with high dose steroids followed by maintenance therapy of steroid sparing DMARDs.

**Disclosure: J. Golla:** None. **E.J. Robinson:** None.

